# Editorial: Psychosocial aspects of adolescents and young adults with cancer

**DOI:** 10.3389/fpsyg.2023.1126571

**Published:** 2023-01-24

**Authors:** Yin Ting Cheung, Andreas Charalambous, Martha Grootenhuis, Ellen van der Plas

**Affiliations:** ^1^School of Pharmacy, Faculty of Medicine, The Chinese University of Hong Kong, Shatin, Hong Kong SAR, China; ^2^Department of Nursing, Cyprus University of Technology, Limassol, Cyprus; ^3^Department of Nursing, University of Turku, Turku, Finland; ^4^Princess Maxima Center for Pediatric Oncology, Utrecht, Netherlands; ^5^Arkansas Children's Research Institute (ACRI), Little Rock, AR, United States

**Keywords:** AYA cancer, psychosocial needs, caregiving-informal, COVID, psychological

Broadly speaking, AYA oncology encompasses clinical care and/or research of individuals diagnosed with cancer between the ages of 15–39 years old. Although varying definitions of the age range are applied, there is no disputing that AYA patients diagnosed or living with cancer (abbreviated as “AYAs” hereafter) experience psychosocial challenges. The AYA period marks a time of tremendous physical-, neurobehavioral-, and social change. Although a continual quest to improve the survival rates of AYA cancers is unquestionably necessary, the unique psychosocial challenges of AYA cancer should also be addressed. This Special Series of *Frontiers in Psychology* highlights research on the unique psychosocial issues faced by AYAs with cancer and their caregivers. Five major topics addressed in this special issue include: (1) the impact of cancer and treatment on AYAs' cognitive, mental, and social well-being, (2) impact of cancer health disparities on psychosocial outcomes among vulnerable groups, (3) informational needs among AYAs, (4) ways to better support family/informal caregivers of AYAs in their caregiving role, and (5) the impact of COVID on the psychosocial outcomes of AYAs.

Focusing on the quality of life aspect of AYA cancer, Tremolada et al. compared cognitive functioning and psychological distress in 205 AYAs and 205 controls. While AYA survivors had lower educational achievement than controls, they reported fewer cognitive problems, and similar psycho-social well-being. Two studies reported on short-term changes in quality of life during the first year of cancer treatment in AYA patients with sarcoma (Day et al.) and young women with breast cancer (Al-Kaylani et al.). Both studies found evidence of short-term improvements in quality of life. Al-Kaylani et al. attribute these early improvements to a sense of relief that may initially outweigh the negative late effects of treatment. These findings underscore the potential importance of identifying AYAs who are at risk of developing psychosocial distress in a clinical setting. The study by Patterson et al. focused on the potential clinical utility and sensitivity of the AYA Psycho-Oncology Screening Tool (AYA-POST). The authors conclude that the AYA-POST can be useful in identifying unique concerns of AYA cancer patients (Patterson et al.). Taken together, these findings suggest that while poor perception of health might be detected in certain subgroups of AYAs during cancer diagnosis and the active treatment phase, their quality of life generally improved during the early post-treatment phase. Since it is still questionable as to whether deficits in quality of life and functioning will emerge during the later survivorship phase, future studies should also evaluate the changes in functional capacity among AYA survivors, such as their work productivity, employment status, and financial security.

Two studies in this series addressed disparity in care (Schwartz-Attias et al.; Kivlighan et al.). The study by Kivlighan et al. focuses on sex-based differences in access to care, where female AYAs were 2.5 times more likely to be referred to behavioral oncology services than male AYAs. The authors call for increased recognition of sex-based biases in referring patients to the appropriate long-term care.

Schwartz-Attias et al. highlight a particularly relevant psychosocial aspect of AYA cancer: health-information found on the internet. The authors' qualitative study revealed that younger AYA cancer patients (15–18 years old) faced challenges in assessing the credibility of the information they received from the internet, and preferred an open discussion with medical professionals. These studies are timely reminders that it is important to consider demographic and sociocultural disparities in AYA psychosocial oncology, especially in developing age-appropriate, gender-specific, and culturally sensitive interventions to address the informational needs and psychological challenges faced by AYAs.

Studies by Melguizo-Garín et al. and Bedoya et al. emphasize the importance of peer/community support in the caregiving journey, and provides important directions in developing family-centered social support interventions for AYAs. Being diagnosed with cancer during a period of significant physical and psychological alterations can create an overwhelming amount of stress to the affected individual, as well as their family/informal caregivers (a role often assumed by a member of their family, companion, or a close friend). Melguizo-Garín et al. demonstrated the positive impact of receiving social support on reducing stress levels in families of AYAs. In line with the notion of “paying it forward,” the same study also found that the act of extending peer support to other families in need, helped to enhance life and family satisfaction among caregivers. When discussions on end-of-life care is inevitable for AYAs who have poor prognosis, Bedoya et al. highlighted the value of having a systematic- and research-informed advance care planning in enhancing open communication among AYAs, family members and friends.

Lastly, the COVID-19 pandemic has created unprecedented challenges in cancer care over the past 3 years. Anxieties related to infection susceptibility, missed appointments due to lockdowns, and uncertainties regarding vaccinations are particularly relevant to AYAs and their caregivers. Nearly half of the AYAs in the study cohort of Hou et al. described that their mental health status as worse now than before the pandemic, while Guido et al. reported that parents with symptoms of post-traumatic stress related to their child's diagnosis appeared to be even more vulnerable to stress symptoms perceived during the pandemic lockdown. Fortunately, the availability of vaccinations and effective viral medications have led to the gradual return to normalcy in most countries/regions. Despite the resolution of this health emergency, there is still an urging need to continuously monitor the traumatic, lasting impact of the pandemic on AYAs and their caregivers.

To conclude, this Special Series presents current evidence and highlights the need for novel research directions on the field of AYA psychosocial oncology. The impact of these psychosocial, cognitive, and behavioral outcomes on AYAs' vocational and/or occupational achievements and functional independence requires further investigation. Future work should aim to address the unique psychosocial needs of AYAs that belong to underserved communities, such as ethnic minorities, LGBTQIA+ AYAs, and individuals from rural communities.

## Author contributions

YC and EP: preparation of manuscript draft. All authors review and editing. All authors contributed to the article and approved the submitted version.

